# The interaction of vasoactive substances during exercise modulates platelet aggregation in hypertension and coronary artery disease

**DOI:** 10.1186/1471-2261-8-11

**Published:** 2008-05-27

**Authors:** Konstantinos Petidis, Stella Douma, Michael Doumas, Ilias Basagiannis, Konstantinos Vogiatzis, Chrysanthos Zamboulis

**Affiliations:** 1 2nd Propedeutic Department of Internal Medicine, Hippokration Hospital, Aristotle University of Thessaloniki, Greece

## Abstract

**Background:**

Acute vigorous exercise, associated with increased release of plasma catecholamines, transiently increases the risk of primary cardiac arrest. We tested the effect of acute submaximal exercise on vasoactive substances and their combined result on platelet function.

**Methods:**

Healthy volunteers, hypertensive patients and patients with coronary artery disease (CAD) performed a modified treadmill exercise test. We determined plasma catecholamines, thromboxane A_2_, prostacyclin, endothelin-1 and platelet aggregation induced by adenosine diphosphate (ADP) and collagen at rest and during exercise.

**Results:**

Our results during exercise showed a) platelet activation (increased thromboxane B_2_, TXB_2_), b) increased prostacyclin release from endothelium and c) decreased platelet aggregation in all groups, significantly more in healthy volunteers than in patients with CAD (with hypertensives lying in between these two groups).

**Conclusion:**

Despite the pronounced activation of Sympathetic Nervous System (SNS) and increased TXB_2 _levels during acute exercise platelet aggregation decreases, possibly to counterbalance the prothrombotic state. Since this effect seems to be mediated by the normal endothelium (through prostacyclin and nitric oxide), in conditions characterized by endothelial dysfunction (hypertension, CAD) reduced platelet aggregation is attenuated, thus posing such patients in increased risk for thrombotic complications.

## Background

Platelet aggregation and thrombosis play a central role in the pathogenesis of atherosclerosis, since platelet aggregates and thrombus formation has been found in the coronary arteries at sites of plaque rupture [1]. Platelets, upon their activation, further stimulate thrombus formation and recruit additional platelets by releasing thromboxane A_2 _(TXA_2_), adenosine diphosphate (ADP), and cytokines, which produce and promote surface thrombin generation and release vasoconstrictor substances [2]. Arterial hypertension, apart from being a major risk factor for atherosclerotic coronary artery disease, is characterized by an increased thrombotic tendency (the thrombotic paradox of hypertension or 'Birmingham paradox') with evidence of platelet activation, hypercoagulation and endothelial dysfunction [3]. Platelet dysfunction has attired attention as a potential cause of increased cardiovascular morbidity and mortality in essential hypertension [4]. All the above suggest the importance of platelet function in coronary artery disease and hypertension.

Platelet function is regulated by several factors that exhibit opposing effects, and the dynamic balance between prothrombotic and anti-antithrombotic factors determines health or thrombosis. Platelet activation is modulated by the function of normal endothelium that exerts a protective antithrombotic effect through the production of nitric oxide (NO) and prostacyclin (PGI_2_), substances that cause vasodilatation and inhibit platelet aggregation [2]. Endothelial dysfunction leads to nitric oxide (NO) deficiency which has been implicated in the underlying pathobiology of many of these disorders (NO insufficiency states) [5]. Moreover, endothelin-1 (ET-1, a potent vasoconscrictive substance released by the endothelium) and plasma catecholamines play a major role in the pathogenesis of acute ischemic events, and stimulate platelet aggregation, thus exerting prothrombotic actions.

In contrast to regular physical exercise that beneficially affects cardiovascular mortality [6], acute vigorous exercise that is characteristically accompanied by sympathetic nervous system (SNS) activation may trigger the occurrence of acute ischemic events [7]. Furthermore, epidemiological studies show that acute ischemic events exhibit a circadian rhythm coinciding with increased activity of the SNS and increased platelet aggregation during early morning hours [8]. SNS activation and the subsequent rise of plasma catecholamines result in increased platelet aggregation (possibly by activating platelet α_2_-adrenergic receptors), thus offering a possible explanation for the association between SNS activation and acute ischemic events [9].

Although several lines of evidence elucidate the function of each one of these vasoactive substances (catecholamines, TXA_2_, prostacyclin, and ET-1) in patients with coronary artery disease, their dynamic interaction remains largely unclarified. The aim of our study was to investigate platelet aggregation during acute submaximal exercise (representing SNS activation) in patients with coronary artery disease and hypertension compared to normal volunteers. Furthermore, we aimed at clarifying the potential contribution of vasoactive substances (such as catecholamines, thromboxane A_2_, prostacyclin and ET-1) on platelet aggregation by the simultaneous determination of these factors during acute exercise.

## Methods

### Study population

Our study population consisted of 60 subjects divided in three groups. Group A consisted of 18 healthy volunteers. They had never experienced signs or symptoms of coronary artery disease, their blood pressure was within normal levels, and they had no other risk factors for atherosclerosis (diabetes mellitus, hypercholesterolemia, obesity, smoking), and no positive family history of hypertension or coronary artery disease.

Group B consisted of 26 patients with essential hypertension (BP > 140/90 mmHg) according to current guidelines (JNC-7 and ESH/ESC). Secondary hypertension was excluded by clinical and, if appropriate, laboratory examination. All patients had recently diagnosed mild or moderate hypertension, confirmed in consecutive visits, and had never used antihypertensive drugs. Hypertensive patients with other cardiovascular risk factors beyond hypertension were excluded.

Group C consisted of 16 patients with known coronary artery disease that were in stable condition for at least three months. All patients were not on antiplatelet medication due to gastrointestinal problems.

Thus, we included three clearly discrete populations (completely healthy individuals without any cardiovascular risk factor, pure hypertensives without additional risk factors, and patients with overt coronary artery disease) in order to avoid potential confounding factors that affect platelet aggregation. The various number of subjects in each group is ought to the difficulties observed in performing study protocol.

Overall 144 subjects were screened and 78 of them were eligible for and agreed to participate in the study. Sixty-six patients were excluded from the study because they had concomitant conditions, such as diabetes mellitus or impaired glucose tolerance (21 patients), hyperlipidaemia (16 patients), smoking (18 subjects), extreme obesity (9 subjects), or positive family history for coronary artery disease or hypertension (10 subjects). The study protocol was very strict and laborious, and several difficulties had to be overtaken; thus, full compliance with protocol procedures was achieved in only 60 out of 78 individuals participating in the study. Specifically, blood sampling during treadmill exercise at all time points was not successful in 8 subjects, platelet aggregation could not be determined in 5 subjects due to technical difficulties, and the simultaneous determination of all vasoactive substances at each time point was not possible in 5 patients due to several technical factors. There were no statistically significant differences in baseline characteristics between the subjects excluded and the subjects participating in the study.

All individuals were sedentary and were not exercising on a regular basis. All subjects gave informed consent and the procedures followed were in accordance with institutional guidelines. The study was approved by the Hospital Ethics Committee and was in accordance with the principles of the Helsinki declaration.

### Study protocol

All medications in the CAD group were stopped for at least one week. The use of aspirin, non-steroid anti-inflammatory drugs or other drugs that interfere with platelet function was prohibited for two weeks.

Blood pressure was measured using a mercury sphygmomanometer, following standard methodology. Three blood pressure recordings were obtained consecutively, and blood pressure was determined as the mean of the second and the third recording. Systolic blood pressure was defined at the point of appearance of the sounds (Korotkoff phase I), and diastolic blood pressure at the point of disappearance of the sounds (Korotkoff phase V).

A treadmill exercise test (Studio 350/4, Kardiolin, Quinton) was performed (modified protocol by Bruce) for 15 minutes with blood pressure and ECG monitoring. All subjects completed the exercise test with no symptoms or ECG signs of myocardial ischemia.

All tests were performed in the morning (8 am). With the patient in the supine position an intravenous indwelling catheter (18G) was inserted and normal saline (0.9%) was infused at a rate of 1 ml/min during the test. Blood was drawn with the ''two syringe" method. Samples were considered suitable for examination if blood flowed freely in the syringe. In total 4 blood samples were drawn. The first with the patient in the supine position just before the treadmill exercise (45 minutes after the placement of the intravenous catheter), the second sample was drawn after the completion of the first stage of the exercise test (9 min), the third sample at the end of the exercise (15 min) and the fourth in the recovery period with the subject in the supine position for 10 min.

### Methods

Platelet aggregation was determined by nephelometry (method by Born). Blood was collected in tubes containing sodium citrate (0.11 M) in 9:1 ratio and centrifuged at 150 × g for 5 min. The resulting platelet-rich (PRP) plasma was kept at room temperature for use within 1 h. The rest of the blood was centrifuged at 1500 × g for 15 min and platelet-poor plasma (PPP) was prepared. Platelet counts were adjusted in each sample with the addition of homologous PPP to 250 ± 50 × 10^9^/L. Platelet aggregation was determined with a four-channel aggregometer (PAP-4 Bio/Data). Platelet aggregation was induced by 50 μl of collagen and 50 μl of ADP to the final concentration of 10 μg/ml of collagen and 1 μM ADP. The aggregometer was adjusted before each experiment so that PRP permitted no light transmittance and the PPP permitted 100% light transmittance. Aggregation was expressed as the maximal percent change in light transmission from baseline, using PPP as a 100% and PRP as 0% reference of aggregation at the end of the recording time. Aggregation curves were recorded for 4 min.

Samples for plasma catecholamine (noradrenaline-NA and adrenaline-ADR) determination were collected in prechiled tubes containing heparin as anticoagulant. Catecholamines were measured with the use of High Performance Liquid Chromatography with Electrochemical Detector (HPLC-ECD).

Samples for plasma ET-1 determination were collected in tubes containing tripotassium ethylenediamine tetra-acetate (K_3_EDTA). Plasma ET-1 concentrations were determined by radioimmunoassay (Nichols Institute Diagnostics, San Juan Capistrano, CA), after solid-phase extraction on C^14 ^columns, evaporation in vacuum-centrifuge, and reconstruction of the solid remainder.

Plasma prostacyclin is highly unstable so we measured the stable product 6-Keto-PGF1a with a radioimmunoassay (DRG Diagnostics). Plasma Thromboxane A_2 _is also unstable, so we measured the stable metabolite TXB_2 _by radioimmunoassay (DRG Diagnostics).

We also determined the ratio of 6-keto-prostaglandin-F1a to thromboxane B_2 _as an index of the balance between antiaggregatory Prostacyclin and pro-aggregatory Thromboxane A_2_.

### Statistical analysis

Analysis was performed using the Statistical Package for Social Sciences (SPSS) 11,5 for Windows (SPSS Inc., Chicago, IL, USA). Results are presented as mean ± standard deviation (mean ± SD). Statistical analyses were carried out using the Independent samples t test and the Mann Whitney U test to compare proportions between 2 different groups, and the 2-tailed paired Student's test and the Wilcoxon Signed Rank Test to compare proportions of the same group in different instants. Values of p <0.05 were considered statistically significant.

## Results

The baseline characteristics of the three groups are presented in Table 1. Normal volunteers were younger, leaner and had lower blood pressure than hypertensives and patients with coronary artery disease.

**Table 1:** Baseline characteristics of patients and healthy volunteers

	Healthy volunteers n = 18	Hypertensives n = 26	CAD patients n = 16
Male/Female	15/3	22/4	13/3
Age (y)	33.9 ± 6.2	38.1 ± 6 *	40.9 ± 3.9 *
BMI kg/m^2^	23.54 ± 2.66	28.35 ± 2.57 *	29.07 ± 4.26 *
SBP mmHg	119.1 ± 8	143.2 ± 14.7 *	138.3 ± 21.9 *
DBP mmHg	79.9 ± 4.8	100.9 ± 4 *	91.4 ± 10.1 *
Heart Rate (/min)	72.7 ± 14.4	82.2 ± 11.9 *	76.4 ± 9.9

* p < 0.05 compared to healthy volunteers

CAD: coronary artery disease

**Figure 1:**
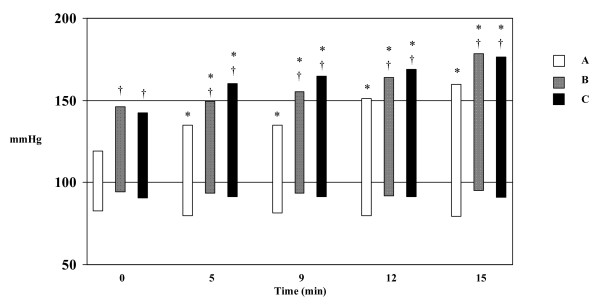
**Blood Pressure** during exercise in healthy volunteers (A), hypertensives (B) and patients with CAD (C). * p<0.05 vs baseline, †p<0.05 vs healthy volunteers.

Systolic blood pressure increased significantly in all three groups during treadmill exercise, while diastolic remained unchanged in patients with coronary artery disease or hypertension and fell, though not significantly, in healthy individuals. Blood pressure at all stages remained higher in the patients with coronary artery disease and hypertension (groups A and B) than in the healthy individuals (Figure 1). The Double Product was determined in all patients at the peak of exercise with no significant differences between the three groups (Table 2). Heart rate at the end of exercise as percentage of the maximum heart rate (predicted by an age based formula; maximum heart rate = 220-age) was determined and there were no significant differences between the three groups (Table 2).

**Table 2:** Characteristics of patients and healthy volunteers during exercise

	Healthy volunteers n = 18	Hypertensives n = 26	CAD patients n = 16
Double Product mmHgxHR/minx10^-3^	26.2 ± 4	28.3 ± 5.4	26.2 ± 4.4
Heart Rate (beats/min)	72.7 ± 14.4	82.2 ± 11.9 *	76.4 ± 9.9
Peak Heart Rate (beats/min)	163.3 ± 14.6	158 ± 17.2	148.3.4 ± 15.7*
Percentage of maximum HR	85%	85.7%	82.8%

* p < 0.05 compared to healthy volunteers in all other parameters no significant differences were found between the three groups

CAD: coronary artery disease

**Figure 2:**
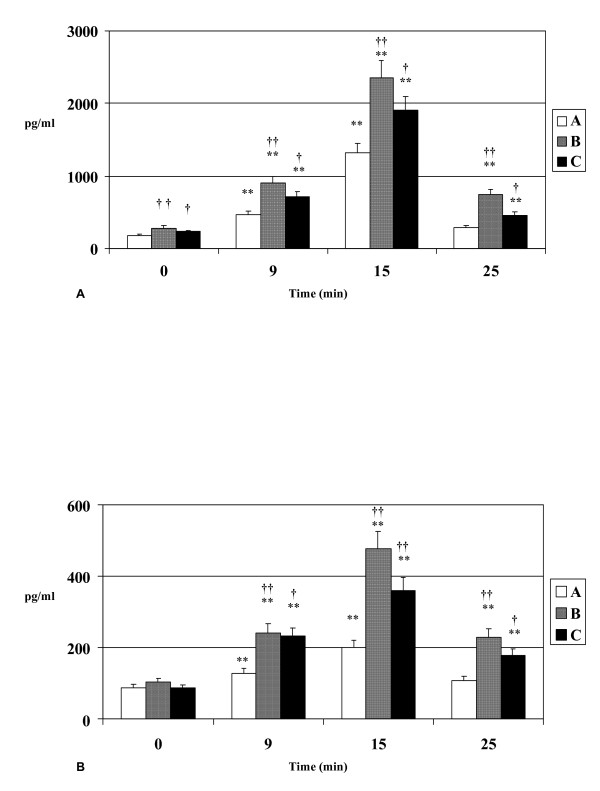
**A. Noradrenaline** levels during exercise in healthy volunteers (A), hypertensives (B) and patients with CAD (C). **B. Adrenaline** levels during exercise in healthy volunteers (A), hypertensives (B) and patients with CAD (C). * p<0.05 vs baseline, **p<0.01vs baseline, †p<0.05 vs healthy volunteers, ††p<0.01 vs healthy volunteers.

Plasma catecholamines increased significantly (p < 0.01) in all groups during exercise. Plasma noradrenaline at rest was higher in hypertensive patients (p < 0.01) and patients with coronary artery disease (p < 0.05) compared to healthy individuals. At exercise peak plasma noradrenaline increased about 10-fold, and hypertensives had higher noradrenaline levels than normal subjects (p < 0.01) and coronary patients (p = non significant, Figure 2A). Plasma adrenaline levels showed a similar fluctuation. At rest, hypertensive patients had higher plasma adrenaline levels but the difference was not significant. Plasma adrenaline increased during exercise in all three groups, and at exercise peak was higher in hypertensive individuals compared to healthy subjects (p < 0.01) and patients with coronary artery disease (p = non significant, Figure 2B).

**Figure 3:**
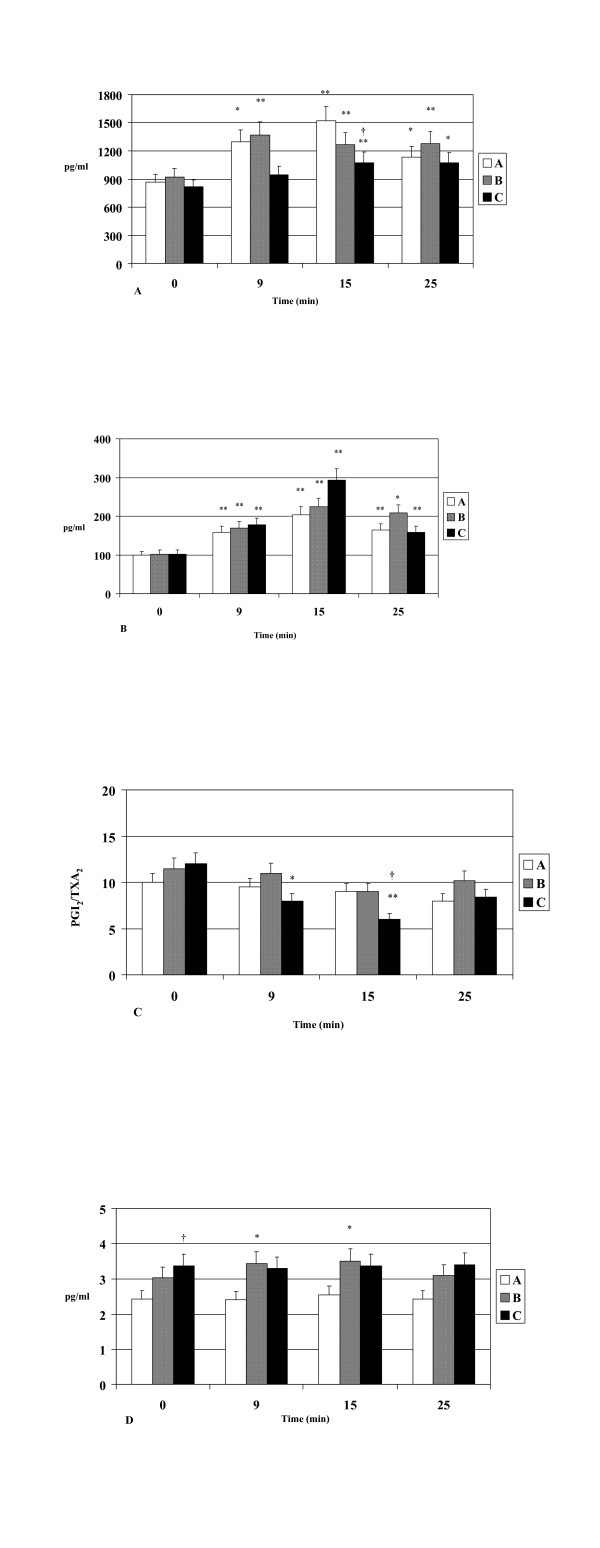
**A. 6-keto-Prostaglandin F1a** levels during exercise in healthy volunteers (A), hypertensives (B) and patients with CAD (C). **B. Thromboxane B2** levels during exercise in healthy volunteers (A), hypertensives (B) and patients with CAD (C). **C. 6-keto-Prostaglandin F1a /Thromboxane B2 (PGI2/TXA2) ratio** during exercise in healthy volunteers (A), hypertensives (B) and patients with CAD (C). **D. Endothelin-1** levels during exercise in healthy volunteers (A), hypertensives (B) and patients with CAD (C). * p<0.05 vs baseline, **p<0.01vs baseline, †p<0.05 vs healthy volunteers, ††p<0.01 vs healthy volunteers.

The 6-keto-prostaglandin-F1a levels increased significantly (p < 0.05) during exercise in all three groups. Patients with coronary artery disease had lower plasma levels of 6-keto-prostaglandin-F1a compared to subjects from the other two groups and the difference was significant only at the peak of exercise compared with healthy volunteers (p < 0.05, Figure 3A).

Plasma TXB_2 _levels showed a significant increase during exercise in all three groups (p < 0.05), reaching their peak at maximum exercise. There was a trend towards higher levels at the end of exercise in patients with coronary artery disease, but there were no significant differences between the three groups (Figure 3B). Plasma TXB_2 _levels remained elevated compared to baseline after 10 min of rest.

The ratio of 6-keto-prostaglandin-F1a to thromboxane B_2 _decreased during exercise in all groups, but the decrease was statistically significant only for the CAD group (p = 0.01). There was no significant difference between the three groups at rest but at the peak of exercise patients with CAD had lower 6-keto-prostaglandin-F1a/thromboxane B_2 _ratio compared with healthy volunteers that almost reached statistical significance (p = 0.053) (Figure 3C). A negative correlation between the PGI_2_/TXB_2 _ratio and platelet aggregation at the end of exercise test with collagen (p < 0.05) and ADP (p = 0.07) was found in the group with CAD compared with controls, indicating that endothelial dysfunction (reduced ratio PGI_2_/TXB_2_) contributes to increased platelet aggregation in CAD patients.

Plasma ET-1 levels were higher at baseline in patients with coronary artery disease compared to healthy volunteers (p < 0.05), while during exercise ET-1 increased significantly (p < 0.05) only in hypertensive patients (Figure 3D).

**Figure 4:**
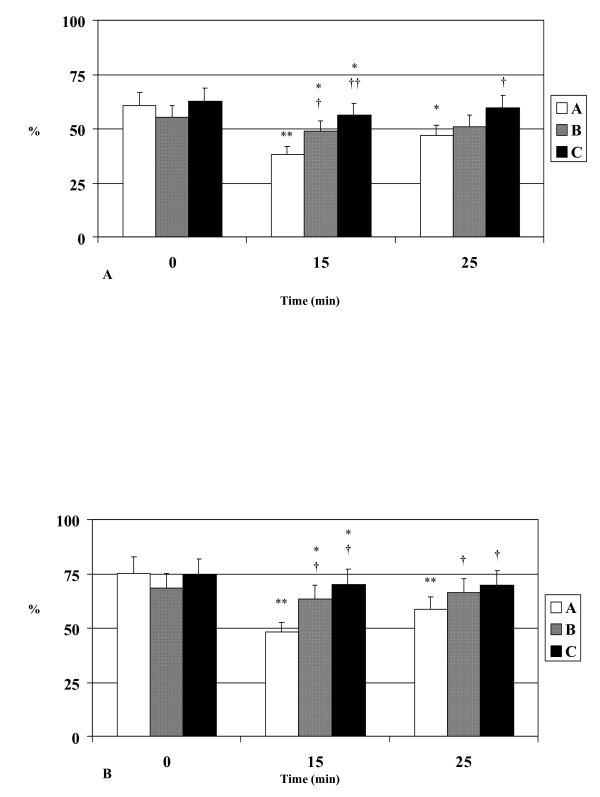
**A. Platelet aggregation (with ADP)** changes during exercise in healthy volunteers (A), hypertensives (B) and patients with CAD (C). **B. Platelet aggregation (with collagen)** changes during exercise in healthy volunteers (A), hypertensives (B) and patients with CAD (C). * p<0.05 vs baseline, **p<0.01 vs baseline, †p<0.05 vs healthy volunteers, ††p<0.01 vs healthy volunteers.

Platelet aggregation decreased significantly during exercise (p < 0.05) in all groups with the use of ADP and collagen as inducing agents. At exercise peak, normal volunteers showed significantly greater inhibition of plasma aggregation when compared with hypertensives (p < 0.05) and patients with coronary artery disease (p < 0.05) with the use of both ADP and collagen (Figure 4A and 4B). After 10 min of rest platelet aggregation remained reduced in healthy volunteers compared to baseline (p < 0.05), while in hypertensives and patients with CAD increased and was higher than in healthy volunteers (p < 0.05).

## Discussion

Thrombotic events represent the most common and important complication of atherosclerotic disease. The clarification of pathophysiologic mechanisms underlying the thrombotic process seems of utmost importance for the prevention of such events and the improved management of these patients. Our study findings indicate that patients with coronary artery disease exhibit a thrombotic tendency compared to healthy subjects during exercise (increased platelet aggregation compared to healthy individuals), while hypertensive patients are lying in between these two groups.

Exercise may induce platelet activation in vivo and enhanced platelet reactivity in vitro, but results concerning exercise-induced platelet aggregation are far from unanimous. Much controversy exists regarding platelet function during SNS activation; studies examining the results of physical and mental stress on platelet aggregation in hypertensives and in patients with ischemic heart disease have shown conflicting results [9-11]. Platelet responses to exercise depend on several factors, such as exercise intensity, the exercise protocol used, and physical fitness. Moreover, measurements of platelet reactivity either in vitro or ex vivo (aggregability assays) or in vivo (platelet secretory products, mainly beta thromboglobulin and platelet factor 4) are associated with considerable methodologic difficulties and thus may account for the discrepancies of results reported in the literature.

Our findings of decreased platelet aggregation during exercise despite the increase in plasma catecholamines seem paradoxical. Increased shear-stress in states of increased SNS activity results in increased platelet aggregation, probably mediated by the action of adrenaline, as its infusion results in increased platelet aggregation in normotensives and hypertensives [12-14]. There is evidence that the increased platelet aggregation in the early morning hours is due to increased sensitivity to the action of adrenaline [15]. The stimulatory effect of epinephrine on platelet functions has been linked to activation of α_2_-adrenoceptor in the membrane of the platelets, since the effect can be blocked by yohimbine, a specific antagonist of α_2_-adrenoceptor [16]; moreover, catecholamines during strenuous exercise have been shown to enhance platelet activation, facilitate the ability of ADP-activated fibrinogen receptors, promote fibrinogen binding to platelet receptors, and more importantly increase the number of α_2_-adrenoceptors despite a decrease in their affinity [17]. Thus, the decreased platelet aggregation found in our study might be explained by two hypotheses: a) either noradrenaline that increases much more than adrenaline (five fold more) during exercise exerts different effects on platelet aggregation, or b) other factors induced by physical exercise counter-regulate the negative effects of adrenaline on platelet aggregation.

Regarding the first hypothesis, experimental studies have shown increased intra-arterial thrombosis during adrenaline infusion but decreased thrombosis during noradrenaline infusion [18]. In addition, prolonged exposure of platelets to increased adrenaline levels results in decreased aggregation to adrenaline, probably due to adrenergic receptor downregulation [19]. There is also evidence that β_2 _adrenergic receptor stimulation causes inhibition of adhesion and aggregation in platelets through increased NOS activity in platelets [20]. β_2 _adrenergic receptor-mediated inhibition of adhesion and aggregation in platelets may partially offset the proaggregatory effects of α_2 _adrenergic receptor stimulation in response to the endogenous catecholamines (noradrenaline and adrenaline).

Regarding the second scenario, noradrenaline has been reported to be able to stimulate endothelial cells to release prostacyclin and nitric oxide, both of which are known to be potent inhibitors of platelet aggregation [21]. In addition, the increased blood flow during exercise causes increased shear stress and vasodilatation through the production of NO and PGI_2_[22]. The increased prostacyclin production, plasma NO metabolites and platelet cGMP may suppress platelet reactivity [23-25]. All our patients exhibited increased PGI_2 _production during exercise, a potent inhibitor of platelet aggregation. The difference was significant between healthy volunteers and patients with coronary artery disease.

Accumulating evidence suggests that platelets of hypertensive patients show signs of activation^25 ^and an increased tendency to aggregate [27]. Hypertensive patients showed higher urinary excretion rate of 11-dehydro-TXB_2 _compared with normotensive controls [28]. Furthermore, exercise is associated with increased TXA_2 _production [29-31]. On the other hand, products of platelet activation (serotonin, TXA_2_, ADP) directly increase platelet aggregation. At the same time, these agents act on endothelial cells and cause further release of prostacyclin and NO and inhibit platelet aggregation^2^. During exercise TXB2 production was increased in all groups, more in patients with coronary artery disease and less in healthy volunteers but the difference was not statistically significant. The balance between PGI_2 _and TXA_2_, as expressed by the ratio of 6-keto-prostaglandin-F1a to thromboxane B_2 _was changed in favour of the prothrombotic TXA_2 _in patients with CAD, that exhibit the higher degree of endothelial dysfunction.

Increased endothelin-1 levels during exercise may contribute to the pathogenesis of acute myocardial infarction that sometimes follows vigorous physical exercise. Even small increments of ET-1 may facilitate vasoconstrictive responses of coronary vessels to other stimuli. Although ET-1 generation de novo requires several hours there is evidence that ET-1 is stored within internal storage vesicles of endothelial cells, and its secretion is a rapid process with the formation and transit time of a vesicle to the plasma taking approximately 10 min. This could explain the abnormal ET-1 synthesis and release during exercise in our patients with hypertension [32]. The fact that only hypertensive patients exhibited an increase in ET-1 is probably explained by the fact that known mechanisms of ET-1 secretion, as increased SNS activity, pressure and shear stress [33] were higher during exercise in hypertensive patients. Experimental data concerning ET-1 effects on platelet function are contradictory showing decreased [34], increased [35] or unchanged [36] platelet aggregation. The increased levels of ET-1 in hypertensives and patients with coronary artery disease are consistent with the endothelial dysfunction present in these patients and the role of ET-1 in cardiovascular disease.

Endothelial dysfunction is characterized by a reduction in prostacyclin and NO, probably resulting in a thrombogenic vascular environment that allows thrombus formation induced by exposure of highly thrombogenic substances from ruptured or erosive plaques. Even though the differences were not statistically significant, healthy individuals had higher PGI_2 _and lower TXA_2 _levels, while patients with coronary heart disease had lower PGI_2 _and higher TXA_2_. The negative correlation between the PGI_2_/TXA_2 _ratio and platelet aggregation in CAD patients indicates that endothelial dysfunction contributes to the thrombotic tendency in these patients.

A major limitation of our study is that normotensives are younger and leaner than the other two groups, and despite their abstinence of regular exercise this may have influenced favourably their response to exercise. Although evidence regarding the relationship between obesity and platelet aggregation are contradictory [37], data from the Northwick study suggest that obesity (body weight or body mass index) is not associated with platelet aggregation in a large sample of about 1000 subjects [38]. Another important limitation of our study is the lack of oxygen consumption estimation during exercise in order to evaluate exercise intensity; however, the use of the double product and the percentage of maximum heart rate achieved at exercise peak as indirect indices of oxygen consumption (revealing a comparable exercise intensity in the three groups) overcomes this limitation. Finally, in our study, NO metabolites and platelet aggregation after adrenaline induction were not determined; we believe that the evaluation of these parameters, in a clinical setting similar to ours, will significantly contribute to the clarification of the mechanisms underlying thrombosis in CAD, thus representing an excellent idea for further research in this area.

## Conclusion

In summary, platelet aggregation was inhibited during exercise in healthy individuals, hypertensives and patients with coronary artery disease. This inhibition of platelet aggregation probably represents a protective endothelial mechanism through the production of PGI_2 _and NO. Endothelial dysfunction is probably responsible for the smaller inhibition of platelet aggregation during exercise in patients with coronary artery disease and hypertensives compared to healthy subjects. The negative correlation between the PGI_2_/TXA_2 _ratio and platelet aggregation in CAD patients indicates that endothelial dysfunction contributes to the thrombotic tendency in these patients. Other causes as defective intraplatelet NO production or resistance of platelets to the action of NO cannot be refuted.

## Abbreviations

CAD: coronary artery disease; TXB_2_: thromboxane B_2_; SNS: Sympathetic Nervous System; ADP: adenosine diphosphate; TXA_2_: thromboxane A_2_; NO: nitric oxide; PGI_2_: prostacyclin; ET-1: endothelin-1; PRP: platelet-rich plasma; PPP: platelet-poor plasma; NA: noradrenaline; ADR: adrenaline; HPLC-ECD: High Performance Liquid Chromatography with Electrochemical Detector;

## Competing interests

The authors declare that they have no competing interests.

## Authors' contributions

KP, SD, MD, IB and CZ participated in the study design and conception and coordination of the project. KP, SD, MD and IB contributed to the provision of participants and study material. KP, SD, MD, KV performed all laboratory tests. KV assembled the data and performed statistical analysis. KP, MD drafted the article which was revised by SD and CZ. All authors read and approved the final manuscript.

## Pre-publication history

The pre-publication history for this paper can be accessed here:


